# Fast decisions reflect biases, slow decisions do not

**Published:** 2024-01-02

**Authors:** Samantha Linn, Sean D. Lawley, Bhargav R. Karamched, Zachary P. Kilpatrick, Krešimir Josić

**Affiliations:** 1 Department of Mathematics, University of Utah, Salt Lake City, Utah, USA; 2 Department of Mathematics, Florida State University, Tallahassee, Florida 32306, USA; 3 Institute of Molecular Biophysics, Florida State University, Tallahassee, Florida 32306, USA; 4 Program in Neuroscience, Florida State University, Tallahassee, Florida 32306, USA; 5 Department of Applied Mathematics, University of Colorado Boulder, Boulder, Colorado 80309, USA; 6 Department of Mathematics, University of Houston, Houston, Texas 77004, USA; 7 Department of Biology and Biochemistry, University of Houston, Houston, Texas 77004, USA

## Abstract

Decisions are often made by heterogeneous groups of individuals, each with distinct initial biases and access to information of different quality. We show that in large groups of independent agents who accumulate evidence the first to decide are those with the strongest initial biases. Their decisions align with their initial bias, regardless of the underlying truth. In contrast, agents who decide last make decisions as if they were initially unbiased, and hence make better choices. We obtain asymptotic expressions in the large population limit that quantify how agents’ initial inclinations shape early decisions. Our analysis shows how bias, information quality, and decision order interact in non-trivial ways to determine the reliability of decisions in a group.

Evidence accumulation models are used widely to describe how different organisms integrate information to make choices [3]. Experimental evidence shows that these models capture the dynamics of the decision making process of humans and other animals, including the tradeoff between speed and accuracy [5, 25, 30, 34, 37]. Such models can also be used to understand how decisions are made in social groups, both when individuals observe each other’s choices [10, 19, 31, 36] and when they act independently [33].

The accumulation of evidence is often modeled using biased Brownian motion with the quality of evidence determining the magnitude of drift and diffusion. An agent is assumed to commit to a decision when the process crosses a threshold. Most previous evidence accumulation models describe a single agent. However, questions remain about how the order of choices in a group is related to their accuracy [40]. In a group of initially unbiased individuals accumulating evidence of different quality, the fastest and most accurate decisions are made by those accessing the highest quality information [31]. Here we ask how the initial biases of individuals in a group impact the order and accuracy of their choices. When is a decision driven mainly by an agent’s initial bias as opposed to accumulated evidence?

We show that in large groups of agents starting with different initial biases, early decisions tend to be made by agents with the most extreme predispositions. The choices of these agents agree with their initial bias, regardless of the quality of the evidence they have access to. On the other hand, decisions of late deciders do not depend on their initial bias. Thus, in large groups early decisions reflect only initial inclinations, regardless of which choice is right. Late decisions reflect only accumulated evidence and are more likely to be correct. These effects hold generically, but not in the special case of initially unbiased agents [31].

## Model description.

**FIG. 1.**
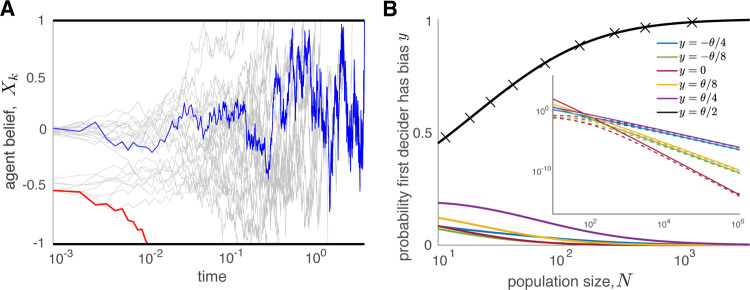
Initial bias determines the choice of early deciders. (A) Evolution of beliefs of *N* = 10^4^ agents who each have even odds of initially being unbiased or biased (*P* (*X*_*j*_(0) = *x*_*i*_) = 0.5, *x*_*i*_ = 0*, −*0.5). The first agent (red) decides according to their initial bias, and makes the wrong decision at *T*_1_ *≈* 0.01. The last agent (blue) decides correctly at *T*_10000_ *≈* 10. (B) Probability that the agent with the largest initial bias decides first as a function of population size, *N*. Solid curves were determined by numerical quadrature (Eq. (S4)) with initial biases assigned with uniform probability from values listed in the legend; black crosses denote results of a stochastic simulation averaged over 10^6^ trials. Inset: Log-log plot of the same results with dashed curves showing the asymptotic results in Eq. (3). Throughout, agents use identical thresholds ±*θ* = ±1, drift *μ* = 1, and diffusivity *D* = 1.

We first assume that each individual in a population of *N* agents has to decide between two choices (hypotheses), *H*^+^ and *H*^*−*^. They do so by accumulating evidence and computing the conditional probabilities, *P* (*H*^±^|evidence), that one of the two hypotheses is correct. When observations are independent and each provides weak evidence, the log likelihood ratio, or *belief*, of agent *i* in the group, *X*_*i*_ = log (*P* (*H*^+^|evidence_*i*_)*/P* (*H*^*−*^|evidence_*i*_)), evolves approximately as a biased Brownian motion [3, 7] (See Fig. 1A),

(1) $$\text{d}X_{i}=\mu_{i}\text{d}t+\sqrt{2D_{i}}\text{d}W_{i},$$

where the drift, *μ*_*i*_, and diffusion coefficient, *D*_*i*_, capture the strength and noisiness of the evidence, respectively [26]. For all agents the correct choice (*H ∈ H*^±^) is given by the sign of the drift (sign[*μ*_*i*_] = ±1). Eq. (1) is widely used and accurately captures the dynamics of decisions in humans and animals, including variability in response time and the impact of evidence quality and biases on choice [8, 23, 30].

Agents start with an initial bias, *X*_*i*_(0), reflecting information or assumptions they have about the prior probability of either hypothesis [23]. We denote by *y* the initial data for a generic agent. Each agent then accumulates evidence, and its beliefs evolve according to Eq. (1). Agent *i* makes a decision when its belief reaches one of two thresholds, −*θ* < 0 < *θ*, at *decision time $\tau_{i}≔\text{inf}\left\{t>0:X_{i}(t)\notin (-\theta ,\theta )\right\}$* This decision, denoted by *d*_*i*_ = *H*^±^, is determined by the sign of the threshold reached, sign[*X*_*i*_(*τ*_*i*_)]. If decision criteria differ between agents an appropriate rescaling of *X*_*i*_(0), *μ*_*i*_, and *D*_*i*_ allows us to assume that all agents use the same thresholds [3].

## Agents with the most extreme initial biases decide first.

We show that in large groups agents whose initial biases are closest to one of the thresholds make the earliest decisions. We first assume observers are identical except for their initial biases, so that *μ*_*i*_ = *μ* and *D*_*i*_ = *D* in Eq. (1). We denote by *T* the *i*^th^ decision time so that *T*_1_ ≤ *T*_2_ ≤ · · · ≤ *T*_*N*_, where *T*_*i*_ = *τ*_*n*(*i*)_ and *n*(*i*) is the index of the *i*^th^ agent to decide. Hence, the index of the first decider is *n*(1).

For simplicity, we assume that each agent starts with one of finitely many initial beliefs, *{ x*_0_*, x*_1_*, … , x*_*I−*1_*}*, sampled with probability *q*_*i*_ = *P* (*X*_*j*_(0) = *x*_*i*_) for *i* = 0*, …, I −* 1. The distance of the initial belief *x*_*i*_ to the closest threshold is *L*_*i*_ = min*{θ − x*_*i*_*, x*_*i*_ + *θ}*. Let *i* = 0 be the index of the unique most extreme initial belief held by an agent, so *L*_0_ < *L*_*i*_ for *i ≠* 0. For a fixed number of initial beliefs, *I*, the first agent to decide in a large group is the one with the largest initial bias (Fig. 1A), in the sense that

(2) $$P\left(X_{n(1)}(0)=x_{0}\right)\to 1\text{ as }N\to \infty \text{. }$$

More precisely, in the Supplemental Material (SM) we show that

(3) $$P\left(X_{n(1)}(0)=x_{i}\right)\sim \eta_{i}{\left(\text{ln }N\right)}^{\left(\beta_{i}-1\right)/2}N^{1-\beta_{i}}$$

as *N* → ∞ for each *i ≠* 0, where

$$\beta_{i}={\left(L_{i}/L_{0}\right)}^{2}>1,$$

$$\eta_{i}=\frac{q_{i}}{q_{0}^{\beta_{i}}}\text{exp}\left(\frac{\sqrt{\beta_{i}}}{2D}\left(\mu_{i}L_{0}-\mu_{0}L_{i}\right)\right)\sqrt{\beta_{i}\pi^{\beta_{i}-1}}\Gamma \left(\beta_{i}\right)>0,$$

and *μ*_*i*_ = ±*μ* if *x*_*i*_ ≷ 0. The same statement holds if *n*(1) is replaced by *n*(*j*) in Eq. (3), but with a change in the prefactor, *η*_*i*_ (See SM). Thus, the probability that the first decision is *not* made by the agent with the most extreme initial belief decreases as a negative power of the population size *N* (Fig. 1B). The approximation given by Eq. (3) is in excellent agreement with the true probabilities when *N* ⪆ 10^3^ (See Fig 1B inset). Moreover, the probability that the agents with the most extreme initial beliefs make the first decision is close to unity already for *N ≈* 100 when initial beliefs are well separated and drift is not too strong.

**FIG. 2.**
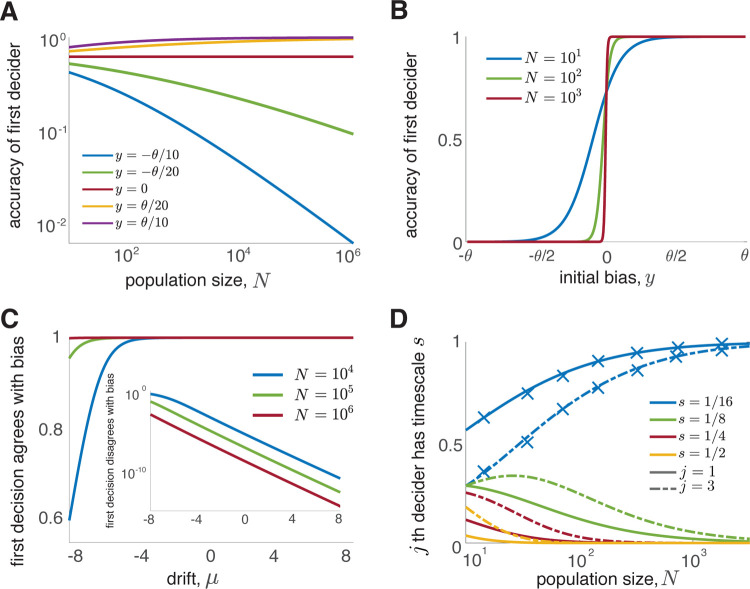
First decider accuracy is determined by its initial bias. (A) The accuracy of the first decider as a function of population size, *N*, for different initial biases, *y*, obtained by quadrature. Curves are ordered by the proximity of the initial bias *y* of the first decider to the correct threshold +*θ*. The drift, and hence the correct decision, are positive. (B) Under the same assumptions a small deviation from an unbiased initial belief strongly affects the probability of a correct first decision when *N* is large. (C) Drift weakly affects the first decision in populations with biased agents (*y* = *θ/*4 here) when *N* is large. See SM for decision polarity formulas. (D) In large populations in which all agents have the same initial bias, *y* = *θ/*2, but different diffusivities, early deciders (here first and third) have the shortest diffusive timescale. X’s represent averages of stochastic simulations over 10^6^ trials.

The choice of the fastest decider agrees with their initial bias: e.g., if *θ* is the threshold closest to the most extreme initial belief, *x*_0_, then *P* (*X*_*n*(1)_(*T*_1_) = *θ*) *→* 1 as *N → ∞* (See Fig. 2A,B). Similar results hold when initial beliefs are drawn from a continuous distribution (See SM and next section). Thus, although all agents behave rationally, early decisions of biased agents tend to be less accurate [11, 33].

In contrast, the probability that a single agent - or one chosen randomly without regard to decision order - decides incorrectly can be made arbitrarily small by increasing the drift or threshold [3]. In large populations with biased agents, drift and diffusion impact the probability of the first decision only through the prefactor in Eq. (3), *η*_*i*_, and thus decrease in importance as population size diverges. If even a small proportion of a large population holds an initial bias, early decisions are determined by the most extreme bias (Fig. 2B) regardless of the drift (Fig. 2C). On the other hand, if all deciders are initially unbiased (*X*_*i*_(0) = 0 for all *i*), the probability the first decider makes a correct choice is (1 + exp(*−μθ/D*))^*−*1^ [3].

## Heterogeneous population and continuous distribution of initial biases.

While we can obtain the most precise asymptotic results in the homogeneous case, our conclusions extend to populations of agents with heterogeneous distributions of initial biases, drifts, diffusivities, and thresholds. We again assume that each agent again starts with one of finitely many initial beliefs, *X*_*i*_(0) *∈ {x*_0_*, x*_1_*, … , x*_*I−*1_*}* with drift and diffusivity sampled from a finite set of fixed size. For each agent we define the diffusive timescale,

(4) $$S_{i}=\frac{L_{i}^{2}}{4D_{i}}>0.$$

By assumption, the timescales *S*_*i*_ follow a discrete distribution P(*S* = *s*_*i*_) > 0 with support on a finite set 0 < *s*_*0*_ ≤ *s*_*1*_ ≤ *s*_*2*_ ≤ *s*_*3*_ ・ ・ ・ ≤ *s*_*J*_, and *S*_*n(j)*_ refers to the timescale of the *j*^*th*^ agent to decide (See Fig. 2D). We denote by s the diffusive timescale of a generic agent.

In large populations, early deciders are those with the shortest diffusive timescales. In particular, we show in the SM that for every *ε* > *0* and fixed *j* ≥ 1,

(5) $$N^{1-s_{1}/s_{0}-\varepsilon }\ll \mathbb{P}\left(S_{n(j)}>s_{0}\right)\ll N^{1-s_{1}/s_{0}+\varepsilon }\text{ as }N\to \infty ,$$

where we use the notation *f ≪ g* to mean lim_*N→∞*_ *f/g* = 0. We can thus conclude that $N^{1-s_{1}/s_{0}-\varepsilon }=o\left(\mathbb{P}\left(S_{n(j)}>s_{0}\right)\right)\text{ and }\mathbb{P}\left(S_{n(j)}>s_{0}\right)=o\left(N^{1-s_{1}/s_{0}+\varepsilon }\right)\text{ as }N\to \infty .$

These results agree with our earlier conclusion: If all agents share the same diffusivity, then the fastest deciders are the agents who start closest to their decision thresholds. This is true regardless of the quality of the evidence they receive. Diffusivity can reduce the effective distance to the threshold according to Eq. (4). Thus, the fastest deciders are either those with the most extreme initial biases or those with the noisiest integration process, regardless of the drift, *μ*_*i*_. Indeed, how we model drift does not impact these conclusions, and they hold even if we model the evolution of beliefs as an Ornstein-Uhlenbeck process, as is frequently done in the psychophysics literature [4, 38].

## Late deciders make decisions as if initially unbiased.

We expect in large populations the inaccuracy of early deciders to be balanced by higher accuracy of late deciders [33]. Thus, we next determine the probability that the last agent to decide makes a correct decision. In the SM we show that this probability has an intuitive form,

(6) $$\mathbb{P}\left(X_{n(N)}\left(T_{N}\right)=\theta \right)\to \int_{-\theta }^{\theta }p_{\theta }(x)q(x)\text{d}x\text{ as }N\to \infty .$$

Here *p*_*θ*_(*x*) is the probability that a single agent with initial bias *X*(0) = *x* makes a correct decision, and *q*(*x*) is the quasi-steady state distribution [27] of beliefs evolving according to Eq. (1). Thus the decision of the last decider is made as if they forget their actual initial bias and instead sample an initial belief from the quasi-stationary distribution, *q*(*x*).

**FIG. 3.**
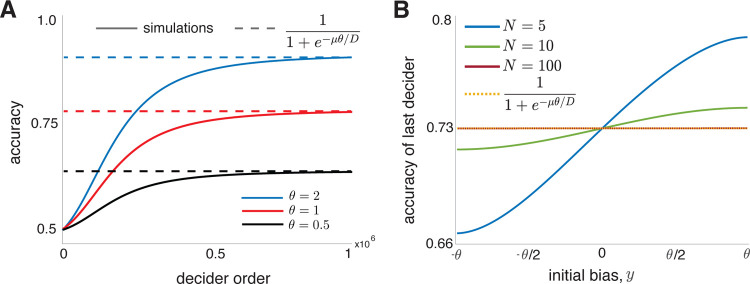
Late deciders make choices as if they held no initial bias. (A) For large *N*, decision accuracy monotonically increases with decision order. The accuracy of late deciders approaches the accuracy of a single, initially unbiased agent. Here, all agents have initial bias *θ/*3, and on each trial, P(*H* = *H*^+^) = 0.5. (B) In large groups even a large initial bias has no impact on the decision of later agents. Here, initial biases are sampled with uniform probability from (*−θ, θ*).

Eq. (6) is general and can be extended to arbitrary domains. When applied to the drift-diffusion process with decision boundaries at ±*θ* we show in the SM that $\mathbb{P}\left(X_{n(N)}\left(T_{N}\right)=\theta \right)\to {\left(1+\text{exp}(-\mu \theta /D)\right)}^{-1}\text{ as }N\to \infty$, which is the probability that a single, initially unbiased decider makes a correct decision (See Fig. 3A) [3]. Thus, the last decider forgets their initial bias and makes decisions based only on the accumulated evidence. The probability that an agent with a large initial bias makes a late decision is small. But should this happen, the initial bias will have little impact on their decision (See Fig. 3B).

## Extension to multiple alternatives.

We can extend these results to decisions between *k* alternatives. Eq. (1) again describes the evolution of beliefs, but now *X*_*i*_(*t*)*, μ*_*i*_ *∈* ℝ^*k −*1^ and *W*_*i*_ is a vector of independent Wiener processes [28]. Each belief evolves on a domain, Ω *⊂* ℝ^*k−*1^, with *k* boundaries [21], each associated with one of the alternatives. Agent *i* chooses alternative *j* if its belief, *X*_*i*_(*t*), crosses the associated boundary first. The boundaries that lead to the best decisions are difficult to find analytically [35], but their exact shape is immaterial for our result.

**FIG. 4.**
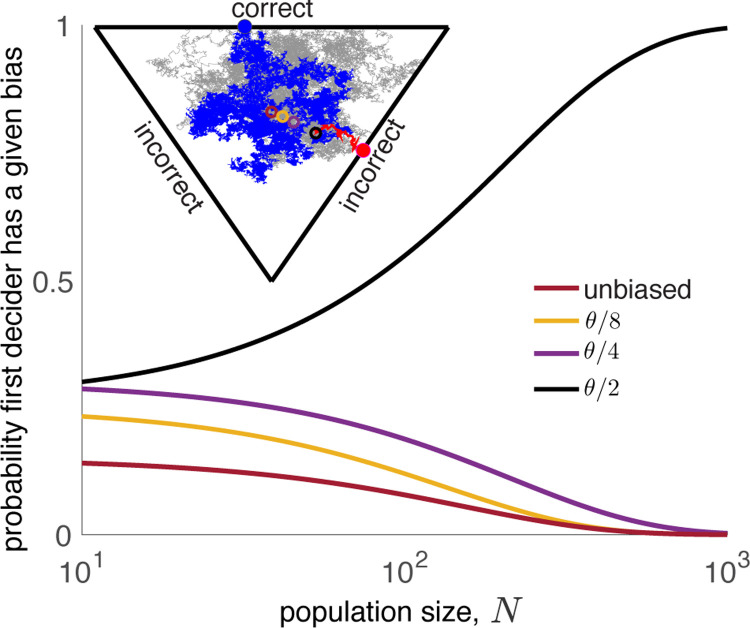
Bias impacts multi-alternative and two-alternative decisions similarly in large groups. Beliefs about three options evolve on an equilateral triangle. Here, *θ* is the closest distance from the center of the triangle (burgundy ring) to the boundary. The initial bias is the distance from the triangle center to the initial belief, *X*_*i*_(0). As *N* increases, the probability that the most biased agent chooses first grows. Curves are computed by averaging 10^6^ stochastic simulations. Inset: Sample trajectories from a trial with biases sampled with equal probability from *{θ/*2*, θ/*4*, θ/*8*}*. The first agent to decide (red) has the largest initial bias. The belief of the last decider (blue) explores the space before reaching a threshold.

In the SM we show that Eq. (3) holds for general domains in arbitrary dimensions (See Fig. 4). We therefore reach our earlier conclusions: In large homogeneous populations, the agents holding the most extreme initial beliefs make the first decisions, and their choices are consistent with their initial biases. Our conclusions about the late decisions also carry over to agents facing multiple choices: The natural extension of Eq. (6) holds with *q*(*x*) the quasi-stationary distribution on Ω. The last decider makes a choice as if it sampled its initial belief from this quasi-stationary distribution.

## Discussion

Our decisions are often influenced by information we obtained previously and predilections we develop. In drift-diffusion models, prior evidence and initial inclinations are often represented by a shift in the initial state. We have shown that initial biases determine early decisions and have a diminishing impact on later decisions.

An agent unaware of the order of their decision would believe this decision was made according to the evidence the agent accumulated and that the accuracy of their choice is determined only by the decision threshold [3]. Though early decisions are not always necessarily less accurate [10], our work identifies a clear case in which hasty choices tend to be the most unreliable. Our findings also suggest a means of weighting choices of biased agents according to decision order in a large group when formulating collective decisions [20]. However, in social groups the exchange of social information between agents [1, 22] or correlations in the evidence [33] will affect these results.

Ramping activity of individual neurons during decision making has been observed across the brain [12, 32] (although see [14]). Such dynamics may reflect the underlying evidence accumulation process preceding a decision and is often modeled by a drift-diffusion process. Decisions are thought to be triggered by the elevated activity of sufficiently many choice-related neurons [39]. Our results suggest that in large neural populations decisions reflect the most extreme initial neural states, rather than the accumulated evidence, if the activity is uncorrelated. Since neural activity is often correlated [6], the effect of such biases could be tempered.

While we have interpreted our results in the context of social decision theory, they apply more generally to independently evolving drift-diffusion processes on bounded domains [17]: In large populations early threshold crossings reflect only the initial states, while late crossings are independent of initial states and reflect the quasi-stationary distribution. Hence, early crossings reflect initial biases providing fast reactions needed for deadlined biophysical processes [9]. If time allows, quorum sensing processes that weight passages by order could be used [13]. Thus, our theory shows how initial biases can be used to implement population level tradeoffs between speed and accuracy.

## Supplementary Material

1
