# Supplementary material for: Fast decisions reflect biases, slow decisions do not
Source: ArXiv. 2024 Jan 2:arXiv:2401.00306v2. Preprint. [Version 2] (PMC10802676)
Supplement: 1 [file NIHPP2401.00306V2-supplement-1.pdf]

Supplemental Material

MATHEMATICAL PRELIMINARIES

Suppose $\{(\tau_n, Z_n)\}_{n\geq 1}$ is an independent and identically distributed (iid) sequence of realizations of the pair of (possibly correlated) random variables (τ, Z). We have in mind that τ is the decision time (or first passage time (FPT)) of some decider whose stochastic evolution of beliefs is denoted by $\{X(t)\}_{t\geq 0}$ and Z is a vector containing information about this decider, such as their random initial position, drift, diffusivity, and decision made. Define the cumulative distribution function (CDF) of τ,

$$F(t) := \mathbb{P}(\tau \leq t).$$

Further, for any event E that is in the σ-algebra generated by Z, define

$$F_E(t) := \mathbb{P}(\tau \leq t \cap E).$$

In words, E is any event for which we can know whether or not it occurred by knowing Z. For example, we are interested in events E like $E = \{X(0) = \theta/2\}$, $E = \{X(0) \leq 0\}$, $E = \{X(\tau) = \theta\}$, etc.

For a given $N \geq 1$, let $n(j) \in \{1, \dots, N\}$ denote the (random) index of the jth fastest decider out of the first N deciders to make a decision. That is, suppose we order the first N FPTs (or first decision times),

$$T_{1,N} \leq T_{2,N} \leq \cdots \leq T_{N-1,N} \leq T_{N,N},$$

where $T_{j,N}$ denotes the jth fastest FPT,

$$T_{j,N} := \min\big\{\{\tau_1, \dots, \tau_N\} \backslash \cup_{i=1}^{j-1} \{T_{i,N}\}\big\}, \quad j \in \{1, \dots, N\}. \tag{7}$$

Then $n(j)$ is such that

$$\tau_{n(j)} = T_{j,N}. \tag{8}$$

In the examples of interest, the FPTs, τ, have continuous probability distributions (i.e. $F(t)$ is a continuous function) so that the event $\tau_{n^*} = \tau_{n'} < \infty$ for $n^* \neq n'$ has probability zero so there is no ambiguity in Eq. (8).

Since we have the sequence $\{(\tau_n, Z_n)\}_{n\geq 1}$, we denote E_n the event E as it pertains to the nth element in the sequence $\{(\tau_n, Z_n)\}_{n\geq 1}$. For example, if $E = \{X(0) = \theta/2\}$, then $E_n = \{X_n(0) = \theta/2\}$. Similarly, $E_{n(j)}$ is the event E as it pertains to $Z_{n(j)}$.

Throughout the Supplemental Material, we use the notation $\int f(t)\,\mathrm{d}g(t)$ to denote the Riemann-Stieltjes integral of a function f with respect to a function g.

Proposition 1. *For any $j \in \{1, 2, \ldots, N\}$ (denoting an agent by the order j of their decision), we have that*

$$\mathbb{P}(E_{n(j)}) = j\binom{N}{j}\int_0^\infty [F(t)]^{j-1}[1 - F(t)]^{N-j}\,\mathrm{d}F_E(t). \tag{9}$$

In the case $j = 1$ (i.e. the fastest decider), Proposition 1 implies

$$\mathbb{P}(E_{n(1)}) = N\int_0^\infty [1 - F(t)]^{N-1}\,\mathrm{d}F_E(t). \tag{10}$$

Since $1 - F$ is a decreasing function, Eq. (10) implies that the short-time behavior of F and F_E determine the large N behavior of $\mathbb{P}(E_{n(1)})$. More generally, Proposition 1 implies that the short-time behavior of F and F_E determine the large N behavior of $\mathbb{P}(E_{n(j)})$ for $1 \leq j \ll N$.

In the case $j = N$ (i.e. the slowest decider), Proposition 1 implies

$$\mathbb{P}(E_{n(N)}) = N\int_0^\infty [F(t)]^{N-1}\,\mathrm{d}F_E(t). \tag{11}$$

Since F is an increasing function, Eq. (11) implies that the large-time behavior of F and F_E determine the large N behavior of $\mathbb{P}(E_{n(N)})$. More generally, Proposition 1 implies that the large-time behavior of F and F_E determine the large N behavior of $\mathbb{P}(E_{n(N-j)})$ for $1 \ll N - j$.

SOME INTEGRAL ASYMPTOTICS

The following proposition is useful for estimating the large N behavior of some integrals of the form in Eq. (9) and was proved in [17] (See Proposition 2 in [17]). Throughout the Supplemental Material, "$f \sim g$" denotes $f/g \to 1$ (e.g., as $N \to \infty$ or as $t \to 0$).

Proposition 2. *Assume $C_+ > C > 0$, $A > 0$, and $p, q \in \mathbb{R}$. Then there exists a $\delta_0 > 0$ so that for all $\delta \in (0, \delta_0]$, we have*

$$\int_0^\delta t^{q-2}e^{-C_+/t}\big(1 - At^p e^{-C/t}\big)^{N-1}\,\mathrm{d}t \sim \eta(\ln N)^{p\beta - q}N^{-\beta} \quad \textit{as } N \to \infty,$$

where

$$\beta = C_+/C > 1, \quad \eta = C^{q-1}(AC^p)^{-\beta}\Gamma(\beta) > 0,$$

and $\Gamma(\beta) := \int_0^\infty z^{\beta-1}e^{-z}\,\mathrm{d}z$ *denotes the gamma function.*

The following result estimates integrals of the form in Eq. (9) for $1 \le j \ll N$ assuming that $F(t)$ and $F_+(t)$ have short-time t behavior that is characteristic of diffusion.

Theorem 3. *Assume* $F(t)$ *and* $F_+(t)$ *are bounded, nondecreasing, continuous from the right, and satisfy*

$$F(t) \sim At^p e^{-C_0/t} \quad \text{as } t \to 0^+, \tag{12}$$

$$F_+(t) \sim Bt^q e^{-C_+/t} \quad \text{as } t \to 0^+, \tag{13}$$

where $C_+ > C_0 > 0$*,* $A > 0$*,* $B > 0$*, and* $p, q \in \mathbb{R}$*. Then for any fixed integer* $j \ge 1$*, we have*

$$j\binom{N}{j}\int_0^\infty [F(t)]^{j-1}[1-F(t)]^{N-j}\,\mathrm{d}F_+(t) \sim \eta(j)(\ln N)^{p\beta-q}N^{1-\beta} \quad \text{as } N \to \infty,$$

where

$$\beta := C_+/C_0 > 1, \quad \eta(j) := B(C_0)^{q-p\beta}A^{-\beta}\Gamma(j)\Gamma(\beta+j) > 0, \tag{14}$$

and $\Gamma(x) := \int_0^\infty z^{x-1}e^{-z}\,\mathrm{d}z$ *denotes the gamma function.*

Notice that the asymptotic behavior found in Theorem 3 as $N \to \infty$ is independent of $j \ge 1$, except for the constant prefactor $\eta(j)$. Further, this prefactor is an increasing function of j and satisfies

$$\eta(j) = \frac{(j-1)!\Gamma(\beta+j)}{\Gamma(\beta+1)}\eta(1), \quad j \ge 1.$$

The asymptotic behavior in Eq. (12)-(13) is typical for diffusion, but computing the prefactors A and B and the powers p and q can be challenging [15]. Indeed, these constants depend on the details of the system (e.g., drift, space dimension, geometry of the domain, etc.). However, the constants in the exponents C_0 and C_+ are more universal and can be obtained in a very general mathematical setting [16]. The following result yields estimates on the fastest deciders when we only know these constants, which is equivalent to knowing the short-time behavior of $F_+(t)$ and $F(t)$ on a logarithmic scale.

Theorem 4. *Assume $F(t)$ and $F_+(t)$ are bounded, nondecreasing, continuous from the right, and satisfy*

$$\lim_{t\to 0^+} t\ln F(t) = -C_0 < 0, \quad \lim_{t\to 0^+} t\ln F_+(t) \le -C_+ < 0, \tag{15}$$

where $C_+ > C_0 > 0$. Then for every $\varepsilon > 0$,

$$j\binom{N}{j}\int_0^\infty [F(t)]^{j-1}[1-F(t)]^{N-j}\,\mathrm{d}F_+(t) = o(N^{1-\beta+\varepsilon}) \quad \text{as } N\to\infty, \tag{16}$$

where

$$\beta := C_+/C_0 > 1.$$

If, in addition, we assume that

$$\lim_{t\to 0^+} t\ln F_+(t) = -C_+ < 0, \tag{17}$$

then for every $\varepsilon > 0$,

$$N^{1-\beta-\varepsilon} = o\biggl(j\binom{N}{j}\int_0^\infty [F(t)]^{j-1}[1-F(t)]^{N-j}\,\mathrm{d}F_+(t)\biggr) \quad \text{as } N\to\infty.$$

The following result estimates integrals of the form in Eq. (9) for $1 \ll N-j \le N$ assuming that $F(t)$ and $f_i(t) = F_i'(t)$ have large-time t behavior that is characteristic of diffusion in a bounded domain.

Theorem 5. *Assume $F(t)\in[0,1)$ is continuous and nondecreasing and $f_i(t)$ is continuous and bounded and*

$$F(t) = 1 - ce^{-\lambda t} + \text{h.o.t.} \quad \text{as } t\to\infty,$$
$$f_i(t) = \lambda c_i e^{-\lambda t} + \text{h.o.t.} \quad \text{as } t\to\infty,$$

where $\lambda > 0$, $c > 0$, $c_i > 0$. Then for any fixed $j \ge 0$, we have that

$$(N-j)\binom{N}{N-j}\int_0^\infty [F(t)]^{N-j-1}[1-F(t)]^j f_i(t)\,\mathrm{d}t \to \frac{c_i}{c} \quad \text{as } N\to\infty.$$

PROOF OF EQ. (3) IN MAIN TEXT

We now apply Theorem 3 to obtain Eq. (3) in the main text. Suppose the belief of each agent evolves independently according to the following stochastic differential equation (SDE),

$$\mathrm{d}X = \mu\,\mathrm{d}t + \sqrt{2D}\,\mathrm{d}W, \tag{18}$$

where $\mu \in \mathbb{R}$ is a constant drift, $D > 0$ is a constant diffusivity, and $W = \{W(t)\}_{t\geq 0}$ is a standard Brownian motion. Define the FPT,

$$\tau := \inf\{t > 0 : X(t) \notin (-\theta, \theta)\},$$

for some threshold $\theta > 0$. Assume that the initial distribution $\mathbb{P}(X(0) = x_i)$ of each agent is a sum of Dirac masses at a finite set of points $\{x_0, x_1, \ldots, x_{I-1}\}$,

$$\mathbb{P}(X(0) = x) = \begin{cases} q_i & \text{if } x = x_i \text{ for some } i \in \{0, 1, \ldots, I-1\}, \\ 0 & \text{if } x \notin \cup_{i=0}^{I-1} x_i. \end{cases},$$

Letting $F_i(t) \equiv F_{X(0)=x_i}(t) = \mathbb{P}(\tau \leq t \cap X(0) = x_i)$, we have that [17]

$$F_i(t) \sim q_i A_i t^{1/2} e^{-C_i/t} \quad \text{as } t \to 0^+, \tag{19}$$

where

$$C_i = \frac{(L_i)^2}{4D},$$

and

$$A_i = \begin{cases} \exp\left(\frac{-\mu L_i}{2D}\right)\sqrt{\frac{4D}{\pi(L_i)^2}} & \text{if } x_i < 0 \\ \exp\left(\frac{\mu L_i}{2D}\right)\sqrt{\frac{4D}{\pi(L_i)^2}} & \text{if } x_i > 0 \\ \left[\exp\left(\frac{-\mu L_i}{2D}\right) + \exp\left(\frac{\mu L_i}{2D}\right)\right]\sqrt{\frac{4D}{\pi(L_i)^2}} & \text{if } x_i = 0, \end{cases}$$

where L_i is the distance to the closest threshold from x_i,

$$L_i = \min\{\theta - x_i, x_i + \theta\}.$$

Further, we assume $0 \in \{0, 1, \ldots, I-1\}$ is the index of the unique starting location closest to a threshold

$$L_0 = \min\{L_0, L_1, \ldots, L_{I-1}\} < L_i \quad \text{if } i \neq 0,$$

then

$$F(t) \sim F_0(t) \quad \text{as } t \to 0^+.$$

We claim that

$$\mathbb{P}(X_{n(1)}(0) = x_0) \to 1 \quad \text{as } N \to \infty, \tag{20}$$

Thus, when N is large the first decider out of many deciders is always the one with the most extreme initial bias. Using the integral representation in Proposition 1 and applying Theorem 3 yields

$$\mathbb{P}(X_{n(1)}(0) = x_i) \sim \eta_i(1)(\ln N)^{(\beta_i-1)/2} N^{1-\beta_i} \quad \text{as } N \to \infty \quad \text{for each } i \neq 0,$$

where

$$\beta_i = (L_i/L_0)^2 > 1,$$

and

$$\eta_i(1) = \begin{cases} \frac{q_i}{q_0^{\beta_i}} \sqrt{\frac{\pi^{\beta_i-1}}{\beta_i}} \Gamma(\beta_i+1) \exp\big(\frac{\sqrt{\beta_i}}{2D}\big(\mu_i L_0 - \mu_0 L_i\big)\big) & \text{if } x_i \neq 0, \\ \frac{q_i}{q_0^{\beta_i}} \sqrt{\frac{\pi^{\beta_i-1}}{\beta_i}} \Gamma(\beta_i+1) \Big[\exp\big(\frac{\sqrt{\beta_i}}{2D}\big(\mu_i L_0 - \mu_0 L_i\big)\big) + \exp\big(\frac{\sqrt{\beta_i}}{2D}\big(-\mu_i L_0 - \mu_0 L_i\big)\big)\Big] & \text{if } x_i = 0, \end{cases}$$

where $\mu_i = \pm\mu$ if $x_i \gtrless 0$.

FIRST DECISION AGREES WITH INITIAL BIAS

The analysis above shows that the first agent to decide in a large group has the most extreme initial bias. We now show the intuitive result that this first decider's decision agrees with their initial bias. Without loss of generality, assume that the most extreme initial bias is negative, $x_0 < 0$. Letting $F_+(t) = \mathbb{P}(\tau \leq t \cap X(\tau) = +\theta)$, we have

$$\begin{aligned} F_+(t) &= \sum_i \mathbb{P}(\tau \leq t \cap X(\tau) = +\theta \,|\, X(0) = x_i) q_i \\ &\sim \mathbb{P}(\tau \leq t \cap X(\tau) = +\theta \,|\, X(0) = x_{i^+}) q_{i^+} \\ &\sim q_{i^+} A_{i^+} t_{i^+}^{p} e^{-C_{i^+}/t} \quad \text{as } t \to 0^+, \end{aligned}$$

where $i^+ \in \{1, \ldots, I\}$ is the index of the starting location closest to $+\theta$. Using the integral representation in Proposition 1 and applying Theorem 3 yields

$$\mathbb{P}(X_{n(1)}(\tau) = +\theta) \sim \eta_{i^+}^{(1)} (\ln N)^{(\beta_{i^+}-1)/2} N^{1-\beta_{i^+}} \quad \text{as } N \to \infty.$$

CONTINUOUS INITIAL BELIEF DISTRIBUTION

In Section , we showed that the first of many deciders have the most extreme initial beliefs in the case that the population has a discrete initial belief distribution. We now generalize

this calculation to the case that the deciders have a continuous initial belief distribution. In particular, suppose that the decider's initial belief (position) has a smooth probability density $\nu(x)$ with support (a,b) with $-\theta < a < b < \theta$. Suppose that

$$\nu(x) \sim (x-a)^{\alpha_a}\nu_a \quad \text{as } x \to a^+,$$
$$\nu(x) \sim (b-x)^{\alpha_b}\nu_b \quad \text{as } x \to b^-,$$

where the coefficients are positive, $\nu_a > 0$, $\nu_b > 0$, and the powers ensure that ν is integrable, $\alpha_a > -1$, $\alpha_b > -1$. In light of (19), suppose that

$$\mathbb{P}(\tau \le t \,|\, X(0) = x) \sim A(x) t^p e^{-C(x)/t} \quad \text{as } t \to 0^+, \quad \text{uniformly for all } x \in [a,b],$$

where

$$C(x) = (L(x))^2/(4D) > 0, \quad L(x) = \min\{\theta - x, \theta + x\},$$

and $A(x) > 0$ for all $x \in [a,b]$.

It follows that

$$F(t) = \mathbb{P}(\tau \le t) = \int_a^b \mathbb{P}(\tau \le t \,|\, X(0) = x)\nu(x)\,\mathrm{d}x$$
$$\sim t^p \int_a^b A(x)\nu(x)e^{-C(x)/t}\,\mathrm{d}x \quad \text{as } t \to 0^+.$$

We thus need to estimate the small time t asymptotics of the integral

$$I := \int_a^b A(x)\nu(x)e^{-C(x)/t}\,\mathrm{d}x,$$

which is an exercise in Laplace's method [2]. If $b > 0$, then for any $\varepsilon \in (0,b)$, we have

$$\int_0^b A(x)\nu(x)e^{-C(x)/t}\,\mathrm{d}x \sim \int_{b-\varepsilon}^b A(x)\nu(x)e^{-C(x)/t}\,\mathrm{d}x$$
$$\sim A(b)e^{-C(b)/t}\nu_b\Gamma(\alpha_b+1)t^{\alpha_b+1} \quad \text{as } t \to 0^+.$$

Similarly, if $a < 0$, then for any $\varepsilon \in (0,|a|)$, we have

$$\int_a^0 A(x)\nu(x)e^{-C(x)/t}\,\mathrm{d}x \sim \int_a^{a+\varepsilon} A(x)\nu(x)e^{-C(x)/t}\,\mathrm{d}x$$
$$\sim A(a)e^{-C(a)/t}\nu_a\Gamma(\alpha_a+1)t^{\alpha_a+1} \quad \text{as } t \to 0^+.$$

Putting this together, we have that if $b > |a|$, then

$$F(t) \sim A(b)\nu_b\Gamma(\alpha_b + 1)t^{p+\alpha_b+1}e^{-C(b)/t} \quad \text{as } t \to 0^+,$$

and similarly if $|a| > b$ or $|a| = b$.

With these estimates, we can apply Theorem 3 to obtain estimates that the fastest decider(s) have extreme initial beliefs. In particular, suppose we want to estimate

$$\mathbb{P}(a + \varepsilon < X_{n(1)}(0) < b - \varepsilon) \quad \text{for some small } 0 < \varepsilon \ll 1,$$

which is the probability that the fastest decider does not have extreme initial beliefs. If we define the event

$$E = \{a + \varepsilon < X(0) < b - \varepsilon\},$$

then using the notation of Section , we have that

$$\begin{aligned} F_E(t) := \mathbb{P}(\tau \le t \cap E) &= \int_{a+\varepsilon}^{b-\varepsilon} \mathbb{P}(\tau \le t \,|\, X(0) = x)\nu(x)\,\mathrm{d}x \\ &\sim t^p \int_{a+\varepsilon}^{b-\varepsilon} A(x)\nu(x)e^{-C(x)/t}\,\mathrm{d}x \quad \text{as } t \to 0^+, \end{aligned}$$

which can be estimated as above using Laplace's method [2]. In particular, if $b > |a|$, then

$$F_E(t) \sim A(b-\varepsilon)\nu(b-\varepsilon)t^{p+1}e^{-C(b-\varepsilon)/t} \quad \text{as } t \to 0^+,$$

assuming $\nu(b - \varepsilon) > 0$, and similarly if $|a| > b$ or $|a| = b$. With this short-time behavior of $F_E(t)$, we can then plug this into Theorem 3 to show that the first deciders have the most extreme initial beliefs.

HETEROGENEOUS POPULATION WITH MULTIPLE ALTERNATIVES

We next consider the generalized case where the beliefs of the agents in the population evolve as processes with (possibly space-dependent) drift, diffusion coefficient, initial position, and even domain (in their own arbitrary space dimension $d \ge 1$). Suppose the belief of the ith decider evolves according to the following d-dimensional SDE,

$$\mathrm{d}X_i = \mu_i(X_i)\,\mathrm{d}t + \sqrt{2D_i}\,\mathrm{d}W_i, \tag{21}$$

where $\mu_i : \mathbb{R}^d \to \mathbb{R}^d$ is a possibly space-dependent drift, $D_i > 0$ is the diffusion coefficient, and $W(t) \in \mathbb{R}^d$ is a standard Brownian motion in d-dimensional space.

Let $L > 0$ denote an agent's (random) shortest distance they must travel to hit the closest target and let $D > 0$ denote the agent's diffusion coefficient. Define the random timescale

$$S = \frac{L^2}{4D} > 0.$$

Suppose that S has a discrete distribution on a finite set

$$0 < s_0 < s_1 < s_2 < s_3 \cdots < s_I,$$

where

$$\mathbb{P}(S = s_i) = q_i > 0, \quad \sum_{i=0}^{I} q_i = 1.$$

Since we have $N \geq 1$ iid agents indexed from $n = 1$ to $n = N$, we let S_n denote the value of S for the nth agent and $S_{n(j)}$ the value of S for the jth fastest to decide.

We have that [16]

$$\lim_{t \to 0^+} t \ln \mathbb{P}(\tau \leq t) = -s_0 < 0, \quad \lim_{t \to 0^+} t \ln \mathbb{P}(\tau \leq t \cap S = s_i) = -s_i < 0.$$

Hence, Proposition 1 and Theorem 4 imply that for any fixed $j \geq 1$ and $i \in \{1, \dots, I\}$ and any $\varepsilon > 0$,

$$N^{1-s_i/s_0-\varepsilon} \ll \mathbb{P}(S_{n(j)} = s_i) \ll N^{1-s_i/s_0^{\varepsilon}} \quad \text{as } N \to \infty, \tag{22}$$

where we use the notation $f \ll g$ to mean $\lim f/g = 0$. That is, in more traditional notation,

$$N^{1-s_i/s_0-\varepsilon} = o\big(\mathbb{P}(S_{n(j)} = s_i)\big) \quad \text{as } N \to \infty,$$
$$\mathbb{P}(S_{n(j)} = s_i) = o\big(N^{1-s_i/s_0+\varepsilon}\big) \quad \text{as } N \to \infty.$$

In the special case that the agents all move in one space dimension and the drifts are spatially constant (but may differ between agents), we can get the constant and logarithmic prefactors on the decay of $\mathbb{P}(S_{n(j)} = s_i)$ as $N \to \infty$.

The result in Eq. (22) says that in a large population if all the agents have the same diffusion coefficient, then the fastest deciders started closest to their decision thresholds (targets). If we allow the diffusion coefficients to vary between agents, then (22) implies that the fastest deciders started close to their decision thresholds and/or they had big diffusion coefficients.

SLOWEST DECIDERS

Suppose the beliefs of the iid agents diffuse in some d-dimensional spatial domain $U \subset \mathbb{R}^d$ and can be absorbed at one of $m \geq 2$ targets $V_0, \dots, V_{m-1}$ and let $\kappa \in \{0, \dots, m-1\}$ indicate which target the decider eventually hits. Here, we will think of the m targets as parts of the $d-1$ dimensional boundary of the domain, and assume that hitting one of the targets triggers a decision. Following [18, 29], suppose the beliefs of the deciders evolve as stochastic process $\{X(t)\}_{t\geq 0}$ that diffuse according to the SDE

$$\mathrm{d}X(t) = -\nabla V(X(t))\,\mathrm{d}t + \sqrt{2D}\,\mathrm{d}W(t), \tag{23}$$

with reflecting boundary conditions. In Eq. (23), the drift term is the gradient of a given potential, $V(x)$, and the noise term depends on the diffusion coefficient $D > 0$ and a standard d-dimensional Brownian motion (Wiener process) $\{W(t)\}_{t\geq 0}$. The survival probability conditioned on the initial position,

$$\mathbf{S}(x,t) := \mathbb{P}(\tau > t \,|\, X(0) = x),$$

satisfies the backward Kolmogorov (also called backward Fokker-Planck) equation,

$$\begin{aligned} \tfrac{\partial}{\partial t}\mathbf{S} &= \mathcal{L}\mathbf{S}, \quad x \in U, \\ \mathbf{S} &= 0, \quad x \in \text{targets}, \\ \tfrac{\partial}{\partial \mathbf{n}}\mathbf{S} &= 0, \quad x \in \text{reflecting boundary (if there is one)}, \\ S &= 1, \quad t = 0. \end{aligned} \tag{24}$$

In Eq. (24), the differential operator $\mathcal{L}$ is the generator (i.e. the backward operator) of Eq. (23),

$$\mathcal{L} = -\nabla V(x) \cdot \nabla + D\Delta,$$

and $\frac{\partial}{\partial \mathbf{n}}$ is the derivative with respect to the inward unit normal $\mathbf{n} : \partial U \to \mathbb{R}^d$.

Using the following weight function of Boltzmann form ,

$$\rho(x) := \frac{e^{-V(x)/D}}{\int_U e^{-V(y)/D}\,\mathrm{d}y}, \tag{25}$$

one can check that the differential operator $\mathcal{L}$ is formally self-adjoint on the weighted space of square integrable functions (see, for example, Ref. [29]),

$$L^2_\rho(U) := \Big\{ f : \int_U |f(x)|^2 \rho(x)\,\mathrm{d}x < \infty \Big\},$$

using the boundary conditions in (24) and the following weighted inner product,

$$(f, g)_\rho := (f, g\rho) = \int_U f(x)g(x)\rho(x)\,\mathrm{d}x,$$

where $(f, g) = \int_U f(x)g(x)\,\mathrm{d}x$ denotes the standard L^2-inner product (i.e. with no weight function). Expanding the solution to (24) yields,

$$\mathbf{S}(x, t) = \sum_{n\geq 1}(u_n, 1)_\rho e^{-\lambda_n t}u_n(x) = \sum_{n\geq 1}(u_n, \rho)e^{-\lambda_n t}u_n(x), \tag{26}$$

where

$$0 < \lambda_1 < \lambda_2 \leq \ldots, \tag{27}$$

denote the (necessarily positive) eigenvalues of $-\mathcal{L}$. The corresponding with eigenfunctions $\{u_n(x)\}_{n\geq 1}$ satisfy the following time-independent equation,

$$-\mathcal{L}u_n = \lambda_n u_n, \quad x \in U, \tag{28}$$

and identical boundary conditions as $\mathbf{S}$. Further, the eigenfunctions are orthogonal and are taken to be orthonormal, which means that

$$(u_n, u_m)_\rho = \delta_{nm} \in \{0, 1\}, \tag{29}$$

where δ_{nm} denotes the Kronecker delta function (i.e. $\delta_{nn} = 1$ and $\delta_{mn} = 0$ if $n \neq m$).

If the initial distribution of an agent has probability measure μ_0,

$$\mathbb{P}(X(0) \in B) = \mu_0(B) = \int_B 1\,\mathrm{d}\mu_0(x), \quad B \subset U, \tag{30}$$

then the FPT τ has survival probability given by

$$S(t) := \mathbb{P}(\tau > t \,|\, X(0) =_{\mathrm{d}} \mu_0) = \int_U \mathbf{S}(x, t)\,\mathrm{d}\mu_0(x),$$

where the condition $X(0) =_{\mathrm{d}} \mu_0$ in the conditional probability merely denotes that $X(0)$ has initial distribution given by μ_0. Hence, we obtain the following representation for the survival probability,

$$S(t) = \sum_{n\geq 1} A_n e^{-\lambda_n t} = \sum_{n\geq 1}(u_n, \rho)(u_n, \mathrm{d}\mu_0)e^{-\lambda_n t}, \tag{31}$$

where the coefficients are given by the following integrals,

$$A_n := (u_n, 1)_\rho \int_U u_n(x)\,\mathrm{d}\mu_0(x), \quad n \ge 1. \tag{32}$$

We have that the FPT τ to one of the targets has CDF

$$\begin{aligned} F(t) = \mathbb{P}(\tau \le t) &= 1 - \mathbb{P}(\tau > t) \\ &= 1 - \sum_{k\ge 1}(u_k, \rho)(u_k, \mathrm{d}\mu_0)e^{-\lambda_k t}, \end{aligned}$$

If

$$p_i(x) = \mathbb{P}(\kappa = i | X(0) = x),$$

then

$$\begin{aligned} F_i(t) := \mathbb{P}(\tau \le t \cap \kappa = i) &= \mathbb{P}(\kappa = i) - \mathbb{P}(\tau > t \cap \kappa = i) \\ &= \mathbb{P}(\kappa = i) - \sum_{k\ge 1}(u_k, p_i\rho)(u_k, \mathrm{d}\mu_0)e^{-\lambda_k t}, \end{aligned}$$

and therefore

$$f_i(t) := F_i'(t) = \sum_{k\ge 1}\lambda_k(u_k, p_i\rho)(u_k, \mathrm{d}\mu_0)e^{-\lambda_k t}$$

Applying Proposition 1 and Theorem 5 yields

$$\mathbb{P}(\kappa_{n(N-j)} = i) \to \frac{(u_1, p_i\rho)}{(u_1, \rho)} = \frac{(u_1\rho, p_i)}{(u_1, \rho)} \quad \text{as } N \to \infty.$$

Now, the solution to the forward Fokker-Planck equation is given by

$$p(x, t) = \mathbb{P}(X(t) = \mathrm{d}x \,|\, \tau > t) = \sum_{k\ge 1} e^{-\lambda_k t}(u_k, \mathrm{d}\mu_0)\rho(x)u_k(x).$$

Hence, $u_1(x)\rho(x)/(u_1, \rho)$ is the quasi-stationary distribution (QSD), $q(x)$, defined by

$$\begin{aligned} q(x) = \lim_{t\to\infty}\mathbb{P}(X(t) = \mathrm{d}x \,|\, \tau > t) &= \lim_{t\to\infty}\frac{\mathbb{P}(X(t) = \mathrm{d}x \cap \tau > t)}{\mathbb{P}(\tau > t)} \\ &= \lim_{t\to\infty}\frac{\sum_{k\ge 1} e^{-\lambda_k t}(u_k, \mathrm{d}\mu_0)\rho(x)u_k(x)}{\sum_{k\ge 1}(u_k, \rho)(u_k, \mathrm{d}\mu_0)e^{-\lambda_k t}} \\ &= \lim_{t\to\infty}\frac{e^{-\lambda_1 t}(u_1, \mathrm{d}\mu_0)\rho(x)u_1(x)}{(u_1, \rho)(u_1, \mathrm{d}\mu_0)e^{-\lambda_1 t}} \\ &= \frac{\rho(x)u_1(x)}{(u_1, \rho)}. \end{aligned}$$

Summarizing, we have shown that

$$\mathbb{P}(\kappa_{n(N-j)} = i) \to \int_U p_i(x)q(x)\,\mathrm{d}x \quad \text{as } N \to \infty. \tag{33}$$

The case of drift-diffusion processes in one dimension. For the one-dimensional example in which all the beliefs of all the agents evolve according to (18), we can compute the QSD, and find that

$$q(x) = \frac{(\pi^2 D^2 + \theta^2\mu^2)\cos(\frac{\pi x}{2\theta})e^{\frac{\mu(\theta+x)}{2D}}}{2\pi D^2\theta(e^{\frac{\theta\mu}{D}}+1)}.$$

Further, it is straightforward to show that the probability that a decider reaches $+\theta$ before $-\theta$ conditioned on the initial belief $x \in [-\theta, \theta]$ is

$$p_1(x) := \mathbb{P}(X(\tau) = +\theta) = \frac{1}{2}\Big(\coth\Big(\frac{\theta\mu}{D}\Big) - 1\Big)e^{\frac{\mu(\theta-x)}{D}}\Big(e^{\frac{\mu(\theta+x)}{D}} - 1\Big)$$

Therefore, applying (33) and explicitly computing the integral yields

$$\mathbb{P}(\kappa_{n(N-j)} = 1) \to \int_{-\theta}^{\theta} p_1(x)q(x)\,\mathrm{d}x = \frac{1}{1+e^{-\frac{\theta\mu}{D}}} = p_1(0) \quad \text{as } N \to \infty.$$

Hence, the slowest deciders out of $N \gg 1$ deciders make a decision as if they were initially unbiased (i.e. as if $X(0) = 0$).

PROOFS

Proof of Proposition 1. Since $\{(\tau_n, Z_n)\}_{n\geq 1}$ are identically distributed, we have that

$$\begin{aligned}\mathbb{P}(A_{n(j)}) &= \sum_{\substack{\text{distinct indices}\\ n_1,\dots,n_N\in\{1,\dots,N\}}} \mathbb{P}(\max\{\tau_{n_1},\dots,\tau_{n_{j-1}}\} < \tau_{n_j} < \min\{\tau_{n_{j+1}},\dots,\tau_{n_N}\} \cap A_{n_j}) \\ &= j\binom{N}{j}\mathbb{P}(\max\{\tau_1,\dots,\tau_{j-1}\} < \tau_j < \min\{\tau_{j+1},\dots,\tau_N\} \cap A_j),\end{aligned} \tag{34}$$

where the coefficient comes from noting that the number of terms in the sum is obtained by choosing the j fastest FPTs out of N and then choosing which of those j will be the jth fastest. Define

$$\tau_j^{(A_j)} = \begin{cases} \tau_j & \text{if } A_j \text{ occurs,} \\ +\infty & \text{if } A_j \text{ does not occur,} \end{cases}$$

so that if $j < N$,

$$\begin{aligned}&\mathbb{P}(\max\{\tau_1,\dots,\tau_{j-1}\} < \tau_j < \min\{\tau_{j+1},\dots,\tau_N\} \cap A_j) \\ &\quad = \mathbb{P}(\max\{\tau_1,\dots,\tau_{j-1}\} < \tau_j^{(A_j)} < \min\{\tau_{j+1},\dots,\tau_N\})\end{aligned}$$

To handle the case $j = N$, we can simply replace $+\infty$ by $-\infty$ in the definition of $\tau_j^{(A_j)}$.

Since $\{\tau_n\}_{n\geq 1}$ are iid, we have that

$$\mathbb{P}(\max\{\tau_1, \dots, \tau_{j-1}\} < t) = \mathbb{P}(\max\{\tau_1, \dots, \tau_{j-1}\} \leq t) = [F(t)]^{j-1},$$

where we have used that $F(t)$ is continuous. Similarly,

$$\mathbb{P}(\min\{\tau_{j+1}, \dots, \tau_N\} > t) = [1 - F(t)]^{N-j},$$

Using that $\{\tau_n\}_{n\geq 1}$ are independent, we have

$$\begin{aligned} G(t) :&= \mathbb{P}(\max\{\tau_1, \dots, \tau_{j-1}\} < t < \min\{\tau_{j+1}, \dots, \tau_N\}) \\ &= \mathbb{P}(\max\{\tau_1, \dots, \tau_{j-1}\} < t)\mathbb{P}(t < \min\{\tau_{j+1}, \dots, \tau_N\}) \\ &= [F(t)]^{j-1}[1 - F(t)]^{N-j}. \end{aligned}$$

Combining the above finally yields

$$\begin{aligned} \mathbb{P}(A_{n(j)}) &= j\binom{N}{j}\mathbb{P}(\max\{\tau_1, \dots, \tau_{j-1}\} < \tau_j^{(A_j)} < \min\{\tau_{j+1}, \dots, \tau_N\}) \\ &= j\binom{N}{j}\mathbb{E}[G(\tau_j^{(A_j)})] \\ &= j\binom{N}{j}\int_0^\infty [F(t)]^{j-1}[1 - F(t)]^{N-j}\,\mathrm{d}F_E(t), \end{aligned}$$

which completes the proof. □

The proof of Theorem 3 is similar to the proof of Theorem 3 in [17].

Proof of Theorem 3. Define the integral from $t = a$ to $t = b$,

$$I_{a,b} := \int_a^b [F(t)]^{j-1}[1 - F(t)]^{N-j}\,\mathrm{d}F_+(t).$$

Let $\varepsilon \in (0, 1)$. By the assumptions in Eq. (12)-(13), there exists a $\delta > 0$ so that

$$A_{-\varepsilon} t^p e^{-C_0/t} \leq F(t) \leq A_{+\varepsilon} t^p e^{-C_0/t} \quad \text{for all } t \in (0, \delta), \tag{35}$$

$$B_{-\varepsilon} t^q e^{-C_+/t} \leq F_+(t) \leq B_{+\varepsilon} t^q e^{-C_+/t} \quad \text{for all } t \in (0, \delta), \tag{36}$$

where $A_{\pm\varepsilon} := A(1\pm\varepsilon)$ and $B_{\pm\varepsilon} := B(1\pm\varepsilon)$. Using Eq. (35) and integrating by parts yields

$$
\begin{aligned}
I_{0,\delta} &\leq \int_0^\delta (A_{+\varepsilon}t^p e^{-C_0/t})^{j-1}(1-A_{-\varepsilon}t^p e^{-C_0/t})^{N-j}\,\mathrm{d}F_+(t) \\
&= (A_{+\varepsilon}\delta^p e^{-C_0/\delta})^{j-1}(1-A_{-\varepsilon}\delta^p e^{-C_0/\delta})^{N-j}F_+(\delta) \\
&+ (N-j)\int_0^\delta (A_{+\varepsilon}t^p e^{-C_0/t})^{j-1}(pt^{-1}+C_0t^{-2})A_{-\varepsilon}t^p e^{-C_0/t}\big(1-A_{-\varepsilon}t^p e^{-C_0/t}\big)^{N-j-1}F_+(t)\,\mathrm{d}t \\
&- (j-1)\int_0^\delta (A_{+\varepsilon}t^p e^{-C_0/t})^{j-1}(pt^{-1}+C_0t^{-2})\big(1-A_{-\varepsilon}t^p e^{-C_0/t}\big)^{N-j}F_+(t)\,\mathrm{d}t \\
&\leq (A_{+\varepsilon}\delta^p e^{-C_0/\delta})^{j-1}(1-A_{-\varepsilon}\delta^p e^{-C_0/\delta})^{N-j}F_+(\delta) \\
&+ (N-j)\int_0^\delta (A_{+\varepsilon}t^p e^{-C_0/t})^{j}(pt^{-1}+C_0t^{-2})\big(1-A_{-\varepsilon}t^p e^{-C_0/t}\big)^{N-j-1}B_{+\varepsilon}t^q e^{-C_+/t}\,\mathrm{d}t \\
&- (j-1)\int_0^\delta (A_{+\varepsilon}t^p e^{-C_0/t})^{j-1}(pt^{-1}+C_0t^{-2})\big(1-A_{-\varepsilon}t^p e^{-C_0/t}\big)^{N-j}B_{-\varepsilon}t^q e^{-C_+/t}\,\mathrm{d}t,
\end{aligned}
\tag{37}
$$

where we have used Eq. (36) in the final inequality. The first term in the righthand side of Eq. (37) vanishes exponentially fast as $N\to\infty$. Using Proposition 2 to find the large N behavior of the second two terms in the righthand side of Eq. (37) and the fact that $I_{\delta,\infty}$ vanishes exponentially fast as $N\to\infty$ yields

$$\limsup_{N\to\infty}\frac{j\binom{N}{j}I_{0,\infty}}{\eta_j(\ln N)^{p\beta-q}N^{1-\beta}} \leq \frac{(1+\varepsilon)}{(1-\varepsilon)^\beta}.$$

The analogous argument yields the lower bound

$$\liminf_{N\to\infty}\frac{j\binom{N}{j}I_{0,\infty}}{\eta_j(\ln N)^{p\beta-q}N^{1-\beta}} \geq \frac{(1-\varepsilon)}{(1+\varepsilon)^\beta}.$$

Since $\varepsilon\in(0,1)$ is arbitrary, the proof is complete. □

Proof of Theorem 4. Define the integral from $t=a$ to $t=b$,

$$I_{a,b} := \int_a^b [F(t)]^{j-1}[1-F(t)]^{N-j}\,\mathrm{d}F_+(t).$$

By Eq. (15), there exists a $\delta>0$ so that

$$e^{-(C_0+\varepsilon)/t} \leq F(t) \leq e^{-(C_0-\varepsilon)/t} \quad \text{for all } t\in(0,\delta), \tag{38}$$

$$F_+(t) \leq e^{-(C_+-\varepsilon)/t} \quad \text{for all } t\in(0,\delta). \tag{39}$$

Using Eq. (38) and integrating by parts yields

$$\begin{aligned} I_{0,\delta} &\le \int_0^\delta e^{-(j-1)(C_0-\varepsilon)/t}\big(1-e^{-(C_0+\varepsilon)/t}\big)^{N-j}\,\mathrm{d}F_+(t) \\ &= F_+(\delta)e^{-(j-1)(C_0-\varepsilon)/\delta}\big(1-e^{-(C_0+\varepsilon)/\delta}\big)^{N-j} \\ &\quad + (N-j)(C_0+\varepsilon)\int_0^\delta e^{-(j-1)(C_0-\varepsilon)/t}t^{-2}e^{-(C_0+\varepsilon)/t}\big(1-e^{-(C_0+\varepsilon)/t}\big)^{N-j-1}F_+(t)\,\mathrm{d}t \\ &\quad - \int_0^\delta (j-1)(C_0-\varepsilon)t^{-2}e^{-(j-1)(C_0-\varepsilon)/t}\big(1-e^{-(C_0+\varepsilon)/t}\big)^{N-j}F_+(t)\,\mathrm{d}t. \end{aligned} \tag{40}$$

The first term in the righthand side of Eq. (40) vanishes exponentially fast as $N\to\infty$. To handle the second term in the righthand side of Eq. (40), note that Eq. (39) implies that

$$\begin{aligned} &\int_0^\delta e^{-(j-1)(C_0-\varepsilon)/t}t^{-2}e^{-(C_0+\varepsilon)/t}\big(1-e^{-(C_0+\varepsilon)/t}\big)^{N-j-1}F_+(t)\,\mathrm{d}t \\ &\quad \le \int_0^\delta e^{-(j-1)(C_0-\varepsilon)/t}t^{-2}e^{-(C_0+\varepsilon)/t}\big(1-e^{-(C_0+\varepsilon)/t}\big)^{N-j-1}e^{-(C_+-\varepsilon)/t}\,\mathrm{d}t. \end{aligned} \tag{41}$$

Since the third term in the righthand side of Eq. (40) is nonpositive, applying Proposition 2 to Eq. (41) and using Eq. (40) and the fact that $I_{\delta,\infty}$ vanishes exponentially fast as $N\to\infty$ completes the proof of Eq. (16).

If Eq. (17) holds, then there exists a $\delta>0$ so that

$$\begin{aligned} e^{-(C_0+\varepsilon)/t} \le F(t) \le e^{-(C_0-\varepsilon)/t} \quad &\text{for all } t\in(0,\delta), \\ e^{-(C_++\varepsilon)/t} \le F_+(t) \le e^{-(C_+-\varepsilon)/t} \quad &\text{for all } t\in(0,\delta). \end{aligned} \tag{42}$$

Using Eq. (42) and integrating by parts yields

$$\begin{aligned} I_{0,\delta} &\ge \int_0^\delta e^{-(j-1)(C_0+\varepsilon)/t}\big(1-e^{-(C_0-\varepsilon)/t}\big)^{N-j}\,\mathrm{d}F_+(t) \\ &= F_+(\delta)e^{-(j-1)(C_0+\varepsilon)/\delta}\big(1-e^{-(C_0-\varepsilon)/\delta}\big)^{N-j} \\ &\quad + (N-j)(C_0-\varepsilon)\int_0^\delta e^{-(j-1)(C_0+\varepsilon)/t}t^{-2}e^{-(C_0-\varepsilon)/t}\big(1-e^{-(C_0-\varepsilon)/t}\big)^{N-j-1}F_+(t)\,\mathrm{d}t \\ &\quad - \int_0^\delta (j-1)(C_0+\varepsilon)t^{-2}e^{-(j-1)(C_0+\varepsilon)/t}\big(1-e^{-(C_0-\varepsilon)/t}\big)^{N-j}F_+(t)\,\mathrm{d}t \\ &\ge F_+(\delta)e^{-(j-1)(C_0+\varepsilon)/\delta}\big(1-e^{-(C_0-\varepsilon)/\delta}\big)^{N-j} \\ &\quad + (N-j)(C_0-\varepsilon)\int_0^\delta e^{-(j-1)(C_0+\varepsilon)/t}t^{-2}e^{-(C_0-\varepsilon)/t}\big(1-e^{-(C_0-\varepsilon)/t}\big)^{N-j-1}e^{-(C_++\varepsilon)/t}\,\mathrm{d}t \\ &\quad - \int_0^\delta (j-1)(C_0+\varepsilon)t^{-2}e^{-(j-1)(C_0+\varepsilon)/t}\big(1-e^{-(C_0-\varepsilon)/t}\big)^{N-j}e^{-(C_+-\varepsilon)/t}\,\mathrm{d}t. \end{aligned} \tag{43}$$

The first term in the righthand side of Eq. (43) vanishes exponentially as $N \to \infty$. Using Proposition 2 to estimate the second two terms in the righthand side of Eq. (43) completes the proof. $\square$

Lemma 6. *For fixed $j \in \{0, 1, \dots\}$, $c > 0$, $\lambda > 0$, and $\delta > 0$, we have that*

$$(N-j)\binom{N}{N-j}\int_{1/\delta}^{\infty}\left[1-ce^{-\lambda t}\right]^{N-j-1}e^{-(j+1)\lambda t}\,\mathrm{d}t \to \frac{1}{\lambda c^{j+1}} \quad \text{as } N \to \infty.$$

Proof of Lemma 6. Changing variables

$$u = 1 - ce^{-\lambda t}, \quad \mathrm{d}u = \lambda c e^{-\lambda t}\,\mathrm{d}t$$

yields

$$\begin{aligned}\int_{1/\varepsilon}^{\infty}\left[1-ce^{-\lambda t}\right]^{N-j-1}e^{-(j+1)\lambda t}\,\mathrm{d}t &= \frac{1}{\lambda c^{j+1}}\int_{1-ce^{-\lambda t}}^{1} u^{N-j-1}(1-u)^j\,\mathrm{d}u \\ &= \frac{1}{\lambda c^{j+1}}\left[\frac{(N-j-1)!j!}{N!} - \int_0^{1-ce^{-\lambda t}} u^{N-j-1}(1-u)^j\,\mathrm{d}u\right],\end{aligned} \tag{44}$$

where we have used that $\int_0^1 u^{a-1}(1-u)^{b-1}\,\mathrm{d}u = \Gamma(a)\Gamma(b)/\Gamma(a+b)$. Since the integral in Eq. (44) vanishes exponentially fast, the proof is complete. $\square$

Proof of Theorem 5. Let $\varepsilon \in (0,1)$. By assumption, there exists $\delta > 0$ so that

$$\begin{aligned}1-(1+\varepsilon)ce^{-\lambda t} \le F(t) \le 1-(1-\varepsilon)ce^{-\lambda t} \quad &\text{for all } t \ge 1/\delta, \\ \lambda(1-\varepsilon)c_i e^{-\lambda t} \le f_i(t) \le \lambda(1+\varepsilon)c_i e^{-\lambda t} \quad &\text{for all } t \ge 1/\delta.\end{aligned}$$

Defining the integral from $t = a$ to $t = b$,

$$I_{a,b} := \int_a^b [F(t)]^{N-j-1}[1-F(t)]^j f_i(t)\,\mathrm{d}t,$$

we therefore have that

$$\begin{aligned}(1-\varepsilon)^{j+1}\lambda c_i c^j \int_{1/\delta}^{\infty}\left[1-(1+\varepsilon)ce^{-\lambda t}\right]^{N-j-1}e^{-(j+1)\lambda t}\,\mathrm{d}t &\le I_{1/\delta,\infty} \\ \le (1+\varepsilon)^{j+1}\lambda c_i c^j \int_{1/\delta}^{\infty}\left[1-(1-\varepsilon)ce^{-\lambda t}\right]^{N-j-1}e^{-(j+1)\lambda t}\,\mathrm{d}t.\end{aligned}$$

Since $I_{0,1/\delta}$ vanishes exponentially fast as $N \to \infty$, Lemma 6 implies that

$$\begin{aligned}\left(\frac{1-\varepsilon}{1+\varepsilon}\right)^{j+1}\frac{c_i}{c} &\le \liminf_{N\to\infty}(N-j)\binom{N}{N-j}I_{0,\infty} \\ &\le \limsup_{N\to\infty}(N-j)\binom{N}{N-j}I_{0,\infty} \le \left(\frac{1+\varepsilon}{1-\varepsilon}\right)^{j+1}\frac{c_i}{c}.\end{aligned}$$

Since $\varepsilon \in (0,1)$ is arbitrary, the proof is complete. $\square$

NUMERICAL SOLUTIONS

Numerical solutions were computed via trapezoidal quadrature on Eq. (9) in Proposition 1. In each set of dynamics, we rescaled the drift-diffusion process on $[-\theta, \theta]$ to the interval $[0, \ell]$. The probability density function for hitting the left boundary in this system is [24]

$$f_0(t) := \frac{\mathrm{d}}{\mathrm{d}t} F_0(t) = \exp\Big(-\frac{\mu x_0}{2D} - \frac{\mu^2 t}{4D}\Big) \frac{D}{\ell^2} \phi\Big(\frac{Dt}{\ell^2}, \frac{x_0}{\ell}\Big), \tag{45}$$

where

$$\phi(s, w) := \begin{cases} \sum_{k=1}^{\infty} \exp(-k^2\pi^2 s) 2k\pi \sin(k\pi w), \\ (4\pi s^3)^{-1/2} \sum_{k=-\infty}^{\infty} (w + 2k) \exp\Big(-\frac{(w+2k)^2}{4s}\Big). \end{cases} \tag{46}$$

The expressions in Eq. (46) are equivalent but have distinct utility: the top expansion converges quickly for large s while the bottom expansion converges quickly for small s. Hence, we utilize both expressions to more accurately compute probabilities associated with slow and fast deciders, respectively.

Integrating Eq. (45) yields

$$F_0(t) = \int_0^t f_0(t')\,\mathrm{d}t' = \exp\Big(-\frac{\mu x_0}{2D}\Big) \Phi\Big(\frac{Dt}{\ell^2}, \frac{x_0}{\ell}\Big)$$

with long- and short-time expansions of $\Phi(s, w)$ given by

$$\begin{aligned} \Phi(s, w) &= \int_0^s \phi(s', w)\,\mathrm{d}s' \\ &= \begin{cases} \sum_{k=1}^{\infty} \Big(1 - \exp[-(b + k^2\pi^2)s]\Big) \frac{2k\pi}{b + k^2\pi^2} \sin(k\pi w), \\ \sum_{k=-\infty}^{\infty} \frac{\mathrm{sgn}(2k+w)}{2} \Big(e^{-\sqrt{\frac{b}{D}}|2k+w|} \mathrm{erfc}\big(\frac{|2k+w|}{\sqrt{4s}} - \sqrt{bs}\big) + e^{\sqrt{\frac{b}{D}}|2k+w|} \mathrm{erfc}\big(\frac{|2k+w|}{\sqrt{4s}} + \sqrt{bs}\big)\Big) \end{cases} \end{aligned}$$

where $b = (\mu\ell/2D)^2$. By symmetry one can determine the corresponding probability density and cumulative distribution functions for hitting the right boundary. Altogether, we acquire long- and short-time expressions for the cumulative distribution function of an agent making a decision,

$$F(t) := F_0(t) + F_1(t).$$

Where numerical solutions are illustrated, we use the short-time expressions of ϕ and Φ for $10^{-10} \leq t \leq 1$ and the complementary long-time expressions for $1 < t \leq 100$, discretizing

each time interval into 10^3 log-spaced points. We consider 10^3 terms in each series expansion. Moreover, we take $\ell = 1$ and unless otherwise stated $D = 1$. Finally, where more than one but finitely many initial beliefs are considered, we scale the probability functions according to the corresponding initial distribution as outlined in Section .

Specific details of figures with numerical solutions are as follows: In Fig 1B we illustrate in color Eq. (10) where $F_E = F$ as defined above with $X_{n(1)}(0) = y$. The black curve, which contains the remaining mass of the total probability, is computed as the sum of the colored curves subtracted from one. In Fig 2A-C we illustrate the probability that the first decider chooses the decision at $X(T_1) = \theta$ conditioned on having a particular initial bias. Hence, by definition of conditional probability, the numerical solutions are produced from quadrature on ratios of Eq. (10) with $F_E = F_1$ in the numerator and $F_E = F$ in the denominator with $X_{n(1)} = y$. The inset of Fig 2C is one minus the outset. In Fig 2D we illustrate Eq. (9) where $F_E = F$ and $S_{n(j)} = s$. In Fig 3B we illustrate the probability that the last decider chooses the decision at $X(T_N) = \theta$ conditioned on having a particular initial bias. Similar to Fig 2B, the numerical solutions are produced from quadrature on ratios of Eq. (11) with $F_E = F_1$ in the numerator and $F_E = F$ in the denominator with $X_{n(N)}(0) = y$.

AGENT-BASED STOCHASTIC SIMULATIONS

a. One-dimensional drift diffusion equation. To test the analytical solutions, we solved Eq. (1) in the main text using the Euler-Maruyama method, which describes the evidence accumulation process preceding binary decisions. In this approximation scheme, the true solution to the stochastic differential equation is approximated by a Markov chain Y constructed by setting $Y_0 = X(0)$ and updating Y according to the iterative scheme

$$Y_{n+1} = Y_n + \mu \Delta t + \sqrt{2D} \Delta W$$

where $Y_n \equiv Y(n\Delta t)$ is the value of the Markov chain after the nth update, and the random variables ΔW are independent and identically distributed Gaussian random variables with mean 0 and variance Δt. The equations were integrated until the value of Y_n exceeded $\pm\theta$.

The temporal discretization, Δt, is user-defined. As N grows, the time to first decision decays slowly. Thus, for large N, Δt must be taken to be sufficiently small for accurate representation of decision dynamics. For simulations here, we chose $\Delta t = 10^{-3}$ for $1 \leq N \leq$

1000. For $N > 1000$, we chose $\Delta t = N^{-1}$.

b. Two-dimensional drift diffusion equation. Decisions between three choices require a drift-diffusion model evolving on a planar domain [21]. Updating the discrete-time approximation of Eq. (21) for each observer (dropping the i subscript) using Euler-Maruyama provides the following iterative scheme

$$Y^1_{n+1} = Y^1_n + \mu^1 \Delta t + \sqrt{2D}\Delta W^1,$$
$$Y^2_{n+1} = Y^2_n + \mu^2 \Delta t + \sqrt{2D}\Delta W^2,$$

where $Y^j_n = Y^j(n\Delta t)$ is the value of the belief after the nth update, the random variables ΔW^j are Gaussian random variables with mean 0 and variance σ^2. Equations are integrated until the vector $\begin{pmatrix} Y^1_n \\ Y^2_n \end{pmatrix}$ departs the triangular domain

$$\{(Y^1, Y^2) | Y^2 < h \ \& \ Y^2 > -2h(3Y^1 + 1) \ \& \ Y^2 > 2h(3Y^1 - 1)\}$$

where $h = 1/2/\sqrt{3}$. Choices of each agent are determined by whether the agent crosses the $Y^2 = h$ or $Y^2 = -2h(3Y^1 + 1)$ or $Y^2 = 2h(3Y^1 - 1)$ boundary. For simulations again we use $\Delta t = 10^{-3}$ for $1 \leq N \leq 1000$ and $\Delta t = N^{-1}$ for $N > 1000$.

For the 2D case in an equilateral triangle, the threshold θ is taken to be equal to the length of the apothem—defined as a line from the center of a regular polygon at right angles to any of its sides. Hence, an unbiased agent begins at the centre of the equilateral triangle. We prescribe initial data for biased agents to be anywhere along an apothem except the centre of the triangle.
